# Spectrum of Clinical Manifestations in Children With *WT1 Mutation*: Case Series and Literature Review

**DOI:** 10.3389/fped.2022.847295

**Published:** 2022-04-15

**Authors:** Patricia Arroyo-Parejo Drayer, Wacharee Seeherunvong, Chryso P. Katsoufis, Marissa J. DeFreitas, Tossaporn Seeherunvong, Jayanthi Chandar, Carolyn L. Abitbol

**Affiliations:** ^1^Division of Pediatric Nephrology, Department of Pediatrics, Holtz Children's Hospital, University of Miami Miller School of Medicine, Miami, FL, United States; ^2^Pediatric Renal Transplantation, Miami Transplant Institute, Jackson Health System, Miami, FL, United States; ^3^Division of Pediatric Endocrinology, Department of Pediatrics, University of Miami Miller School of Medicine, Miami, FL, United States

**Keywords:** *Wilms tumor*, congenital nephrotic syndrome, steroid-resistant nephrotic syndrome, 46XY sex reversal, disorders of sexual development

## Abstract

**Background:**

Mutations of the *Wilms tumor suppressor-1 gene* (*WT1)* are associated with life-threatening glomerulopathy, disorders of sexual development, Wilm's tumor, and gonadal malignancies. Our objectives were to describe the clinical presentations, age of progression, and onset of complications of *WT1 mutation* through a case series and literature review.

**Methods:**

A retrospective study included all patients followed at the University of Miami/Holtz Children's Hospital from January 2000 to December 2020 with a diagnosis of *WT1 mutation*. A literature review of *WT1 mutation* cases was analyzed for clinical manifestations, karyotype, and long-term outcomes.

**Results:**

The *WT1 mutation* was identified in 9 children, median age at presentation of 0.9 years (range 1 week to 7 years). A total of four had female phenotypes, and 5 had abnormalities of male external genitalia, while all had XY karyotypes. All progressed to end-stage kidney disease (ESKD) and received a kidney transplant at a median age of 5 years (1.5–15 years). During a median time of follow-up of 9 years (range 2–28 years), there were 2 allograft losses after 7 and 10 years and no evidence of post-transplant malignancy. From 333 cases identified from the literature review, the majority had *female phenotype* 66% (219/333), but the predominant *karyotype was XY* (55%, 183/333). Of the female phenotypes, 32% (69/219) had XY sex reversal. Wilm's tumor occurred in 24%, predominantly in males with gonadal anomalies.

**Conclusions:**

Early recognition of *WT1 mutation* is essential for comprehensive surveillance of potential malignancy, avoidance of immunosuppressants for glomerulopathy, and establishing long-term multidisciplinary management.

## Introduction

*Wilms tumor suppressor gene-1 (WT1*) mutations are associated with life-threatening nephropathy including congenital or/ infantile nephrotic syndrome (CNS) and/or childhood onset steroid-resistant nephrotic syndrome (SRNS) as well as a spectrum of disorders of sexual development (DSD), Wilm's tumor and gonadal malignancies. SRNS is one of the most common causes of end-stage kidney disease (ESKD) in children and adolescents with up to 25% of cases associated with a genetic abnormality preferentially expressed in the podocytes (1, 2). Traditional syndromic terminology includes the terms of *Denys-Drash Syndrome*, which represents early-onset nephrotic syndrome, Wilm's tumor, and male pseudo-hermaphroditism as well as *Frasier Syndrome* with late-onset SRNS and gonadal dysgenesis.

The *WT1* gene is localized on chromosome 11p13 and encodes a transcriptional factor of the zinc-finger protein family. The advances in genetic diagnostic tests have increased the ability to identify genetic causes of childhood SRNS. The *WT1* gene is a tumor suppressor gene necessary for the development of the kidney and gonads. In the fetal kidney, WT1 protein is highly expressed in the areas of active glomerulogenesis, which supports a major role of the gene in the development and maturation of the glomerular filtration barrier (2). Germline heterozygous *WT1 mutations* are known to manifest as the developmental genital abnormalities including 46XY sex reversal with complete female phenotype and various anomalies of the male genitalia such as cryptorchidism and hypogonadism (3, 4). In the recent years, the consensus has been to move away from these terms since the spectra of clinical and phenotypic diseases overlap (5). The risk of Wilm's tumor and gonadoblastoma in individuals with *WT1 mutations* is ominous and merits close surveillance for yet an undetermined period. Therefore, early recognition of *WT1* mutation is critical for the early detection of neoplasia and counseling regarding sexual development and reproductive health. A number of international studies have described the incidence and prevalence of this rare disease and case reports, with comprehensive reviews on the clinical manifestations of *WT1* gene mutations in the pediatric population (6–10).

Our objectives through this case series and contemporary literature review were to describe the clinical presentation, which includes extra-renal manifestations, age of diagnosis, progression, and complications of *WT1* glomerulopathy. We also sought to develop a paradigm for earlier recognition and genetic diagnosis through the phenotypic correlations.

## Methods

A retrospective chart review of patients followed at the University of Miami/Holtz Children's Hospital from January 2000 to December 2020 with a diagnosis of *WT1* gene mutation was performed. Data collection that included age at presentation, sex, ethnicity, karyotype, initial presentations of kidney and extrarenal disease, age at diagnosis of *WT1 mutation*, at ESKD, and at transplantation as well as complications related to *WT1 mutation* that includes malignancy and gonadal disorders was recorded.

A comprehensive literature search and review for original articles and case reports with *WT1 mutation* and glomerulopathy published from 2000 to August 2021 was performed through PubMed. The core concept was to identify information on the spectrum of clinical manifestations and diagnosis of *WT1 mutation*. More specifically, sex, karyotype, and significant complications that include type of malignancy were extracted when available. The two authors independently performed the literature search using keywords: *WT1 mutation, Denys-Drash, Frasier, genotype, CNS, SRNS, glomerulopathy, or nephropathy*. The abstracts of all selected articles were reviewed, and references from retrieved articles were used to identify other relevant sources. To make data extraction accurate and consistent, the studies that did not specify *clinical manifestations* or *karyotype* were excluded. Only articles in the English language were reviewed.

This study was approved by the institutional review board of the University of Miami with the waiver of informed consent and protection of privacy in compliance with the *Health Insurance Portability and Accountability Ac*t (HIPAA).

### Statistical Analyses

All data were analyzed with descriptive statistics with values reported as the median with range for non-parametric data. Normality was determined by the D'Agostino & Pearson test. Odds ratios for risk of malignancy were determined by Fisher's exact test with the 95% confidence intervals (95% CI) reported. Statistical analyses were performed using the GraphPad Prism version 9.12 for Windows, GraphPad Software, La Jolla California USA, www.graphpad.com. *p-value* < 0.05 was considered as statistically significant.

## Results

### Case Series of *WT1* Glomerulopathy

*Wilms tumor suppressor-1 gene* mutation associated with glomerulopathy was identified in nine children (Table 1), of whom four were Hispanic, three Caucasian, one Asian, and one Middle Eastern. The median age at renal presentation was 0.9 years (range 1 week to 7 years). Of the 9 cases, 4 presented as CNS in the first year of life. Two phenotypic males presented with Wilms tumor at <2 years of age. The first (Patient #6) had proteinuria and hypertension early after birth and developed Wilm's tumor at 5 months of age. The second (Patient #8) developed Wilm's tumor at 2 years of age with proteinuria and CKD 3 years later. Two presented with SRNS at 3 and 5 years of age and had received multiple immunosuppressive treatments before diagnosis of *WT1 mutation*. Only one (Patient #4) presented with advanced kidney failure and hypertension at 7 years of age.

**Table 1:** Case series at university of Miami.

**Subject**	**Phenotype**	**Karyotype**	**Ethnicity**	**Initial Presentation**	**Complications of *WT1 mutation***	**Age at onset (years)**	**Age at genetic diagnosis (years)**	**Age at KT (years)**	**Age at last FU (years)**
1	Female	XY	White	CNS	Gonadal dysgenesis ESKD	0.1	15.5	1.5	30
2	Female	XY	White	CNS	Gonadal dysgenesis ESKD	0.5	5.5	5.7	17
3	Female	XY	Hispanic	SRNS	Gonadal dysgenesis ESKD	5.0	15.3	14.5	26
4	Female	XY	Hispanic	ESKD	Gonadal dysgenesis ESKD	7.0	8.0	8.5	13
5	Male^*^	XY	Hispanic	CNS	Cryptorchidism, ESKD	0.1	4.1	4.1	13
6	Male	XY	White	Hypospadias, Proteinuria, Wilm's tumor	Wilms tumor, DSD, ESKD	0.1	0.5	3.5	12
7	Male	XY	Middle East	CNS	DSD, ESKD	0.9	4.1	4.1	6
8	Male	XY	Hispanic	Hypospadias, Proteinuria, Wilm's tumor	Wilms tumor, DSD, ESKD	2.0	5.0	15.3	26
9	Male	XY	Asian	Ambiguous genitalia, SRNS	DSD ESKD	3.0	12.0	13.0	18
*N* = 9	4 Female 5 Male	9 XY	3 White 4 Hispanic 2 Other	4 CNS 2 SRNS 2 Wilm's Tumor 1 ESKD	4 Sex Reversal 5 Male DSD 2 Wilms Tumor 9 ESKD	0.9 (0.1–7)	5.5 (0.5–15)	5.7 (1.5–15)	17 (6–30)

* *Father had childhood SRNS, but not ESKD; data presented in median (range). ESKD, end-stage kidney disease; KT, kidney transplantation; FU, follow-up; CNS, congenital–infantile nephrotic syndrome; SRNS, steroid-resistant nephrotic syndrome; DSD, disorder of sexual development*.

Of the nine subjects, all had 46XY karyotype, but only five had male phenotype with various degrees of abnormal male genital development with ambiguous genitalia (*N* = 1), cryptorchidism (*N* = 4), and hypospadias (*N* = 2). The other four subjects had complete sex reversal and presented as female phenotype. Among the four phenotypic females, two had delayed diagnosis of WT1 *mutation* at 15 years of age when XY karyotype was discovered as a component of the workup for delayed puberty. This was despite their presentation early in life with CNS and childhood onset SRNS, respectively. The other two phenotypic females had genetic diagnosis during the evaluation of SRNS with microarray analysis which revealed the XY karyotype at 2 and 7 years of age, respectively. All phenotypic female patients underwent prophylactic gonadectomy after diagnosis of *WT1 mutation* and have required long-term hormonal replacement. Each of the five phenotypic males has a disorder of sexual development (DSD), which ranges from hypospadias (2), cryptorchidism (2) to ambiguous genitalia with penoscrotal hypospadias, and bilateral cryptorchidism (1) requiring multiple urological surgeries. During the observation period, two males entered puberty, one had hypogonadism, and all continue to be monitored for gonadal tumor and sexual development and function. The median time from renal presentation to diagnosis of *WT1 mutation* was 7.6 years for the phenotypic female patients and 2.2 years for males. Although those of female phenotype tended to be older at genetic diagnosis, none of the age milestones comparing male vs. female phenotypes were significantly different in this small case series.

In our case series, eight of the nine subjects had bilateral nephrectomy before or at the time of transplantation. Those with CNS may have had bilateral nephrectomy because of heavy proteinuria. Those with known *WT1 mutation* likely had native nephrectomy at the time of transplantation as a prophylactic measure. There was no specific protocol regarding the recommendation for prophylactic nephrectomy. However, all phenotypic 46XY females underwent prophylactic gonadectomy after *WT1 mutation* was diagnosed while males with DSD did not.

All progressed to ESKD and received a kidney transplant at a median age of 5 years (range 1.5 to 15 years) with a median follow-up of 9 years (range 2–28 years). There were two allograft losses after 7 and 10 years and no evidence of post-transplant malignancy.

### Results From Literature Review

**Figure 1:**
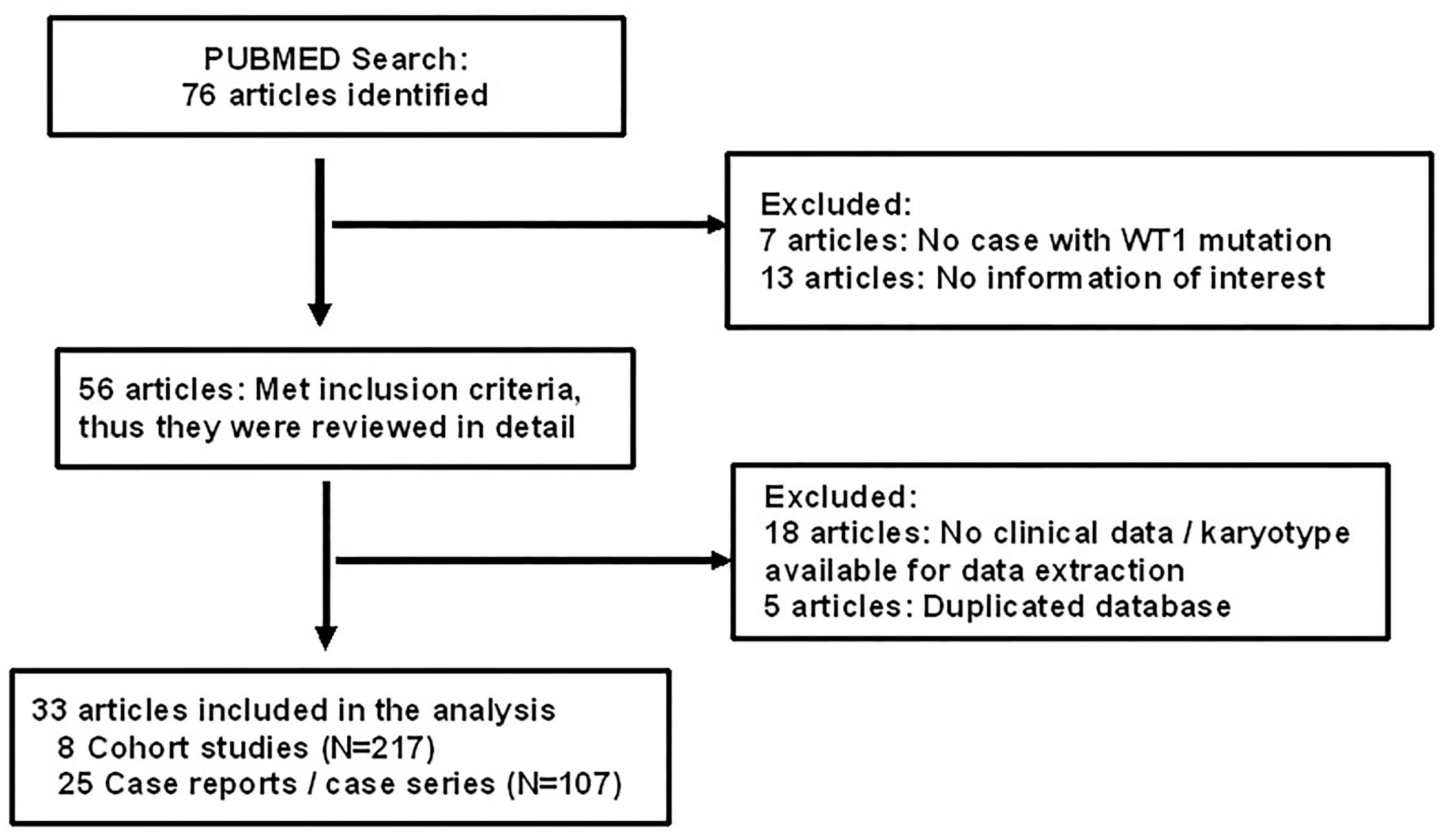
Flow chart showing identification and selection of cases having *WT1* glomerulopathy with data on age, karyotype, and phenotype.

Of 76 articles identified with keywords, 43 were excluded due to the lack of *WT1* glomerulopathy cases, the lack of clinical data on sex/karyotype, age at presentation, or duplicated database (Figure 1). Thus, 33 articles were eligible for comprehensive review. A number of eight were cohort studies of subjects with *WT1* glomerulopathy from various countries (6–8, 10–14) and 25 were from case series or case reports (15–39).

There were 324 subjects extracted from the literature review and 9 from our case series for a total of 333 cases of *WT1* glomerulopathy. The details of phenotype/karyotype, age at presentation, renal and extrarenal presentations, and complications are summarized in Tables 2, 3.

**Table 2:** Compiled data on age of onset, presentation, and complications in *WT1* glomerulopathy.

**Reference**	***N***	**Age at presentation (Years)**			**Initial Clinical Presentation**				**Complication**		
		**<1**	**1–12**	**>12**	**CNS**	**Proteinuria/ SRNS**	**Wilms tumor**	**CKD/ ESKD**	**ESKD**	**Wilms tumor**	**Gonadal tumor**
Denamur et al. (15)	1	0	1	0	0	1	0	0	1	0	0
Ohta et al. (17)	2	2	0	0	2	2	2	2	2	2	0
Takata et al. (16)	27	8	19	0	7	13	0	12	24	2	0
Ito et al. (18)	2	0	2	0	0	1	0	1	2	0	0
Melo et al. (19)	1	0	0	1	0	0	0	1	0	0	1
Auber et al. (20)	12	5	7	0	5	4	8	3	9	8	1
Saylam and Simon (21)	1	0	1	0	0	1	0	1	1	0	0
Hu et al. (22)	2	0	2	0	0	0	0	2	2	2	0
^*^Ruf et al. (11)	8	1	7	0	0	6	1	0	6	1	2
^*^Aucella et al. (12)	4	0	4	0	0	3	0	1	4	0	0
Love et al. (23)	1	0	1	0	0	1	0	0	1	0	1
Gwin et al. (24)	4	1	2	1	1	2	0	1	3	0	4
Bache et al. (25)	1	1	0	0	0	0	0	1	1	0	0
^*^Chernin et al. (6)	38	19	17	2	9	14	7	14	26	12	3
Kohler et al. (27)	7	2	3	2	2	2	2	1	4	2	0
Megremis et al. (26)	4	1	3	0	1	3	0	0	2	0	0
Aydin et al. (30)	1	1	0	0	1	0	0	0	1	1	0
Guaragna et al. (31)	2	0	2	0	0	2	0	0	1	0	0
Yang et al. (28)	1	0	1	0	0	1	0	0	0	0	0
Yang et al. (29)	1	0	1	0	0	1	0	0	1	0	0
Binczak-Kuleta et al. (32)	2	1	1	0	1	1	0	0	0	0	0
^*^Lipska et al. (7)	61	18	42	1	17	55	8	9	46	23	3
^*^Lehnhardt et al. (8)	50	22	28	0	0	45	14	13	39	14	1
Kumar et al. (33)	3	0	3	0	0	3	0	0	2	0	2
^*^Ahn et al. (10)	20	10	10	0	8	8	0	4	19	1	1
Dabrowski et al. (34)	1	1	0	0	0	0	1	0	1	1	0
Chiba and Inoue (35)	1	0	1	0	0	1	0	0	0	0	0
Nishi et al. (36)	7	7	0	0	7	0	0	0	8	1	0
Roca et al. (37)	5	3	2	0	3	1	0	1	5	2	0
Matsuoka et al. (38)	1	0	1	0	0	1	0	0	0	0	1
^*^Nagano et al. (13)	17	7	10	0	7	17	0	9	9	0	0
^*^Sun et al. (14)	33	18	15	0	18	15	0	0	29	3	0
Nagano et al. (39)	3	3	0	0	3	3	0	3	3	0	0
Arroyo-Parejo Drayer, this study	9	4	5	0	3	3	2	1	9	2	0
Total	333	135	191	7	95	210	45	80	205	81	19

* Designates cohort studies. References (6–8, 10–39). ^*^Cohort studies; CNS, congenital–infantile nephrotic syndrome; SRNS, steroid-resistant nephrotic syndrome; CKD, chronic kidney disease; ESKD, end-stage kidney disease.

**Table 3:** Karyotype–phenotype and associated Wilms and gonadal tumors in *WT1* glomerulopathy.

**Reference**	**Country**	**N**	**46 XX**	**46 XY**	**46 XY**			**Wilms tumor/ Phenotype/ Karyotype**			**Gonadal Tumor/ Phenotype/ Karyotype**	
					**F-XY**	**DSD**	**Normal**	**F-XX**	**M-XY**	**F-XY**	**M-XY**	**F-XY**
Denamur et al. (15)	France	1	0	1	0	1	0	0	0	0	0	0
Ohta et al. (17)	Japan	2	2	0	0	0	0	2	0	0	0	0
Takata et al. (16)	Japan	27	7	20	11	2	7	2	0	0	0	0
Ito et al. (18)	Japan	2	1	1	0	1	0	0	0	0	0	0
Melo et al. (19)	Brazil	1	0	1	0	1	0	0	0	0	1	0
Auber et al. (20)	France	12	4	8	1	7	0	4	4	0	1	0
Saylam and Simon (21)	Italy	1	0	1	1	0	0	0	0	0	0	0
Hu et al. (22)	Australia	2	0	2	0	2	0	0	2	0	0	0
^*^Ruf et al. (11)	USA, Europe	8	5	3	1	2	0	1	0	0	0	1
^*^Aucella et al. (12)	Italy	4	2	2	2	0	0	0	0	0	0	0
Love et al. (23)	USA	1	0	1	1	0	0	0	0	0	0	1
Gwin et al. (24)	USA, Spain	4	0	4	4	0	0	0	0	0	0	4
Bache et al. (25)	France	1	0	1	1	0	0	0	0	0	0	0
^*^Chernin et al. (6)	Mixed	38	25	13	4	7	2	6	6	0	0	3
Kohler et al. (27)	Germany	7	0	7	0	7	0	0	2	0	0	0
Megremis et al. (26)	Greece	4	3	1	1	0	0	0	0	0	0	0
Aydin et al. (30)	Turkey	1	0	1	0	1	0	0	1	0	0	0
Guaragna et al. (31)	Brazil	2	2	0	0	0	0	0	0	0	0	0
Yang et al. (28)	China	1	1	0	0	0	0	0	0	0	0	0
Yang et al. (29)	China	1	0	1	0	0	1	0	0	0	0	0
Binczak-Kuleta et al. (32)	Poland	2	1	1	1	0	0	0	0	0	0	0
^*^Lipska et al. (7)	Europe	61	34	27	9	17	1	8	14	1	0	3
^*^Lehnhardt et al. (8)	German	50	19	31	6	24	1	7	10	1	0	1
Kumar et al. (33)	India	3	0	3	2	0	1	0	0	0	0	2
^*^Ahn et al. (10)	Korea	20	8	12	8	4	0	0	1	0	0	1
Dabrowski et al. (34)	USA	1	0	1	0	1	0	0	1	0	0	0
Chiba and Inoue (35)	Japan	1	0	1	1	0	0	0	0	0	0	0
Nishi et al. (36)	Japan	7	2	5	3	2	0	0	1	0	0	0
Roca et al. (37)	Spain	5	2	3	0	3	0	1	1	0	0	0
Matsuoka et al. (38)	Japan	1	0	1	1	0	0	0	0	0	0	1
^*^Nagano et al. (13)	Japan	17	11	6	0	2	4	0	0	0	0	0
^*^Sun et al. (14)	China	33	18	15	7	7	1	2	1	0	0	0
Nagano et al. (39)	Japan	3	3	0	0	0	0	0	0	0	0	0
Arroyo-Parejo Drayer, this study	USA	9	0	9	4	5	0	0	2	0	0	0
Total		333	150	183	69	96	18	33	46	2	2	17

* *Designates cohort studies. References (6–8, 10–39)*.

### Phenotype–Karyotype and Genital Abnormalities

**Figure 2:**
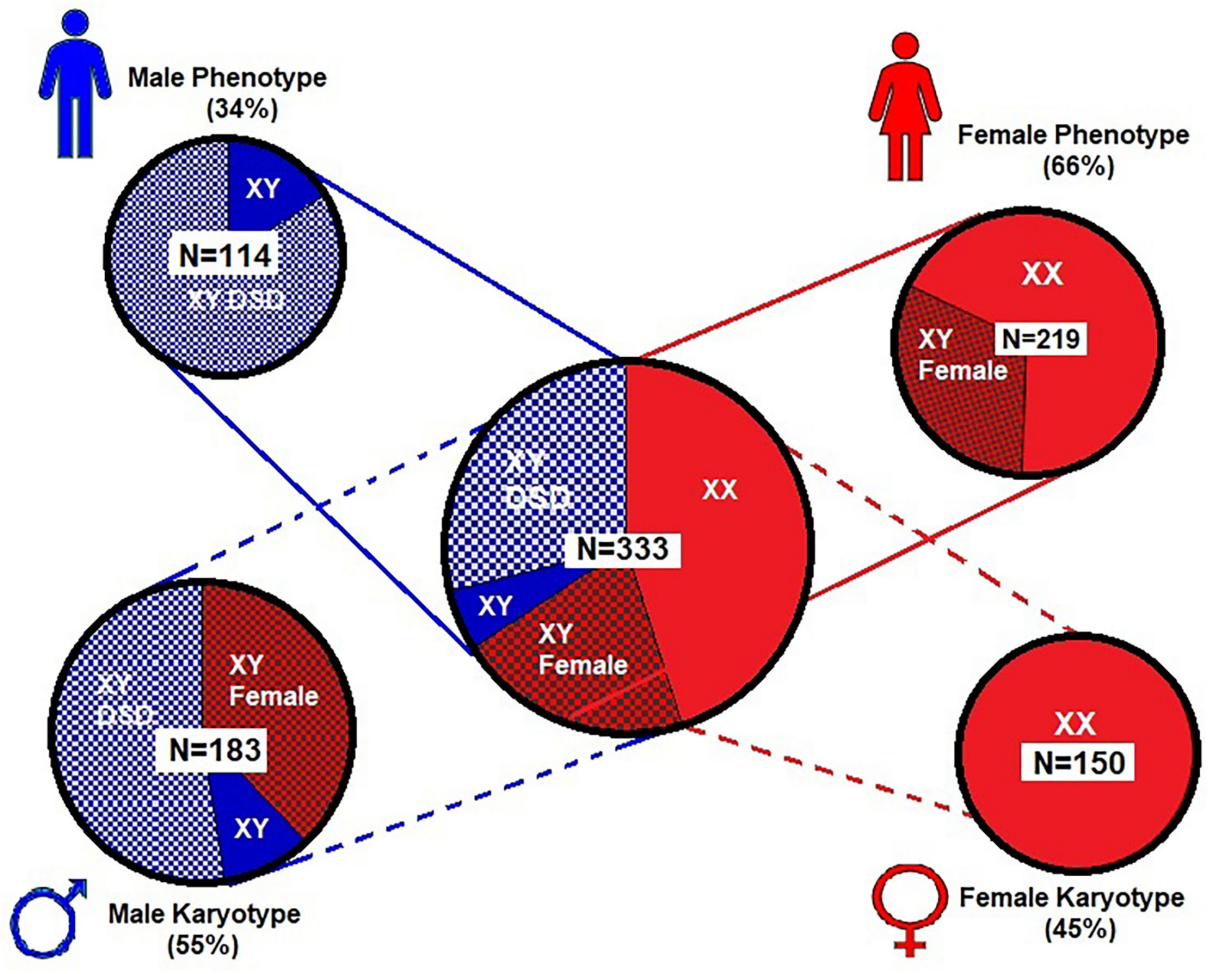
Proportional distribution of phenotype–karyotype for 333 subjects from literature review and our case series. Female (XX) karyotype is only 45% of the entire series while the male (XY) karyotype predominates (55%) and includes predominantly males with disorders of sexual development (DSD). However, the female phenotype was nearly two times that of the male phenotype and includes those with 46 XY sex reversal. For graphic clarity, the phenotype is represented by the male and female iconic figures in blue and red, respectively 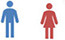. The karyotypes are graphically represented by the male and female gender icons in blue and red, respectively 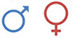.

Figure 2 shows the phenotype–karyotype distribution of the 333 cases of the *WT1 mutation* compiled in the literature review and case series. Of the 333 subjects, the predominant ***karyotype*** was ***XY*** (*N* = 183; 55%). In contrast, the majority ***phenotype*** was ***female*** with 219/333 (66%) including the 69/219 (32%) having XY sex reversal. Of note, the ***male phenotypes*** were in the minority at 114/333 (34%). Of those with the male phenotype, 84% (96/114) had a DSD including hypospadias, cryptorchidism, and/or ambiguous genitalia.

### Renal Presentation and Progression to ESKD

Most cases of *WT1 mutation* (98%) had clinical presentation before 12 years of age. Renal presentation that includes CNS, SRNS, isolated proteinuria, chronic kidney disease (CKD), or ESKD occurred across all age groups. Up to 41% presented in the first year of life with CNS, proteinuria, ESKD, and/or Wilm's tumor. Males often received early recognition with abnormal genitalia at birth but subsequently developed proteinuria and/or Wilm's tumor. Females from 46 XY sex reversal were more likely to have late diagnosis due to delayed puberty.

### Malignancy

The risk of malignancy relative to phenotype–karyotype was analyzed among the entire cohort. Of the 333 subjects, 29% (98/333) developed a malignancy which included 81 (24%) with Wilm's tumor and 98 (29%) with any tumor including Wilm's or gonadal tumor. Wilm's tumor usually presented early before 2 years, and rarely after 5 years of age.

**Figure 3:**
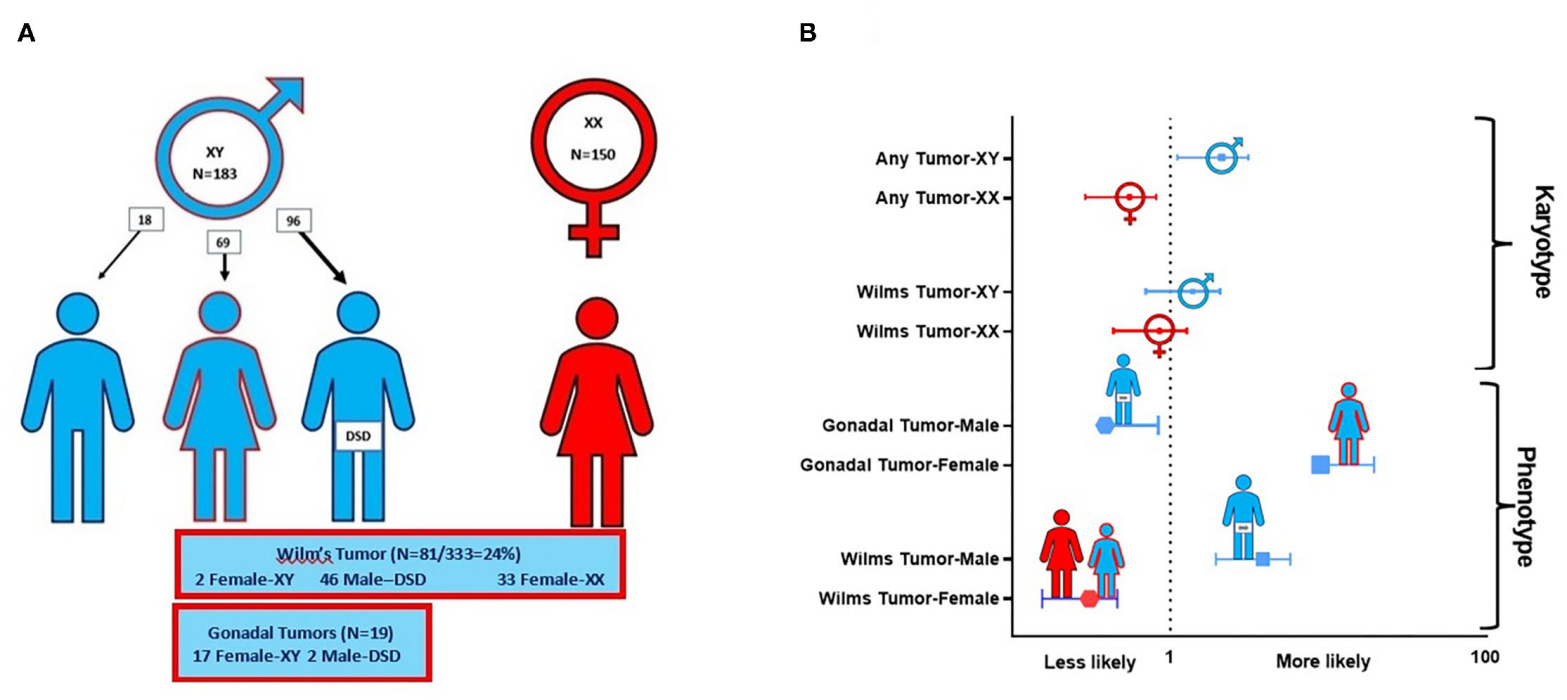
This composite depicts the risks of malignancy in *WT1 mutation* according to phenotype and karyotype. **(A)** Distribution of karyotype and Wilm's tumor and gonadal tumors in 333 cases of *WT1 mutation*. Note that the true male XY (*n* = 18) without DSD developed ***no*** malignancy. Only those with XY karyotype and gonadal dysgenesis (male XY-DSD and female-XY) developed gonadal tumors. **(B)** Risks by odds ratios for developing Wilm's tumor and/or gonadal tumor by karyotype and phenotype in 333 cases of *WT1 mutation*. For graphic clarity, the phenotype is represented by the male and female iconic figures in blue and red, respectively 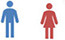. The karyotypes are graphically represented by the male and female gender icons in blue and red, respectively 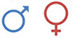.

Figure 3 and Table 4 demonstrate the proportion and odds ratios of subjects who developed malignancy relative to phenotype and karyotype. The male karyotype (XY) was predominant with 183/333 = 55% of the cohort. This included the females with 46 XY sex reversal who comprised 69/183 = 38% of the male karyotype. Those with male karyotype, regardless of phenotype (male-or-female XY), had an odds ratio for developing any malignancy including Wilm's or gonadal tumor of 1.95, 95% CI: 1.2→ 3.2; *p* < 0.01. The major risk of malignancy was with the male phenotype, the majority of whom (96/114 = 84%) had a DSD while only 18 had normal male genitalia (18/114 = 16%). None of the normal males developed a malignancy. Those with male karyotype had a higher but non-significant risk of developing Wilm's tumor as compared to the female karyotype. The XY karyotype odds ratio was 1.26 (95% CI: 0.8→ 2.1) while those with female karyotype had a reciprocal odds ratio of 0.79 (95% CI: 0.5→ 1.3; *p* = 0.44) for the development of Wilm's tumor which was not significant. Overall, those with male phenotype including largely those with DSD had an odds ratio of 3.56 (95% CI 2.1→ 5.9; *p* < 0.0001) of developing Wilm's tumor. Those with female phenotype including the 46 XY sex reversal females had a reciprocal odds ratio of 0.28 (95% CI 0.2→ 0.5; *p* < 0.0001) for developing Wilm's tumor. Gonadal tumors occurred only in XY males with DSD (*n* = 2) and 46 XY females with sex reversal (*n* = 17).

**Table 4:** Online: Odds ratios for risks of malignancy in *WT1 mutation* according to phenotype–karyotype in a case series of 333 subjects.

	**Karyotype/ Phenotype**	**Odds ratio**	**95% CI**	***p*** **-value**	**% of Total tumors (*N* = 91)**	**% of Total population (*N* = 333)**
**Any tumor (Wilm's or Gonadal) by Karyotype (*N* = 91 Tumors)**						
Karyotype	XY	1.95	1.20–3.20	**<0.01**	66%	20%
	XX	0.51	0.31–0.83		34%	10%
**Wilm's tumor by karyotype (*N* = 81 Wilm's tumors)**						
Karyotype	XY	1.26	0.77–2.13	0.4413	59%	14%
	XX	0.79	0.47–1.30		41%	10%
**Wilm's tumor by phenotype (*N* = 81 Wilm's tumors)**						
Phenotype	Male	3.56	2.10–5.90	**<0.0001**	57%	14%
	Female[Table-fn TN4]	0.28	0.17–0.47		43%	11%
**Gonadal tumor by phenotype (*N* = 19 Gonadal tumors)**						
Phenotype	Male	0.21	0.04–0.87	**0.025**	11%	0.6%
	Female[Table-fn TN4]	4.71	1.15–20.9		89%	5.1%

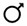
*Includes XY female phenotypes. Bold p-values indicated statistical significance*.

## Discussion

With this case series and extensive literature review, we have compiled important clinical, sex, and karyotype characteristics and projected long-term outcomes of 333 cases with an estimated 500 cases reported worldwide since it was first described in 1991 (5, 40). Although considered an extremely rare disease (41), the broad spectrum of clinical manifestations has become more apparent, and it is suspected that many cases may go unrecognized (8, 27, 42, 43).

Proteinuria is the most frequent and pervasive manifestation of the *WT1 mutation* from birth through childhood to adulthood (5, 8, 42, 43). It occurred in 92% of the 333 cases at presentation in our current review, which is consistent with the previous reviews (8, 42, 43). The progression to ESKD is also synchronous with proteinuria. The concurrence of CNS with renal failure is a frequent clinical presentation in infancy, whereas SRNS with slower progression to ESKD occurs later in childhood.

In this case series and literature review, we were unable to refine the clinical phenotypes with the specific *WT1 mutation* analyses which have become more available in the recent years. These studies have been able to categorize the various mutations with broader clinicopathologic correlations (39). Over 30 mutations or missense variants of the WT1 protein have been analyzed, reported, and correlated with phenotypes that include proteinuria, ESKD, genital anomalies, and Wilm's tumor (13). Subjects with exon 6-9 *WT1* missense mutations display partial gonadal dysgenesis and late-onset nephropathy. The intron 9 splice-site mutations with the 3 amino acid residues–lysine (K), threonine (T), and serine (S)–referred to as the +KTS is an important element in gonadal development and the *WT1* variant that affects the +KTS/-KTS ratio of the isoforms to <2.0 results in abnormal gonadal development with sexual reversal (female XY-DSD) phenotype and late-onset nephropathy. Importantly, the XY sex reversal with female phenotype appears to be protective against the development of Wilm's tumor while it is associated with high risk for gonadoblastoma (19, 44, 45). Although rare, there were 2 cases of Wilm's tumor with female XY sex reversal in this case series (7, 8).

Malignancy is the most ominous complication of the *WT1 mutation* and its occurrence relative to the clinical phenotypic–genotypic profile was delineated by the analysis of the 333 cases in our review. Importantly, Wilm's tumor with *WT1 mutation* tends to occur early with a median age of 15–19 months vs. a median age of 36 months in children without a pathogenic *WT1* variant (5). Any early-onset Wilm's tumor or bilateral Wilm's tumor should have *WT1 mutation* analysis. The 20-year registry of Wilm's tumor has shown that 1.3% of those with unilateral and 15% with bilateral Wilm's tumor progressed to ESKD (46). Hence, this mutation should be considered in toddlers, as well as in cases of bilateral Wilm's tumor (5). A recent single center retrospective analysis of 25 long-term Wilm's tumor survivors with *WT1* pathogenic variants confirmed male predominance (60%), early age occurrence (median 14 months), multiple genetic variants, high incidence of bilateral disease (52%), and genitourinary malformations (44%) (47). This recent report emphasized the importance of early genetic diagnosis to better inform surgical management toward the preservation of renal mass when oncologic management is not compromised. Importantly, our report is the first to describe long-term outcomes of those with *WT1 mutations* post-transplantation. It is notable that we report a cumulative of over 170 patient years with no evidence of post-transplant malignancy.

The complete sex reversal with 46 XY female phenotype is associated with late-onset SRNS, and CKD has been typically categorized as “Frasier syndrome.” These patients are often not diagnosed until adolescence when they fail to achieve puberty. Importantly, this unique phenotype is associated with the KTS gene variant that seems to impart “protection” against the development of Wilm's tumor. It does not prevent gonadoblastoma because the gonads are dysplastic. As in our series, 2 phenotypic females presented with CNS and childhood SRNS, but they were not diagnosed with *WT1 mutation* until adolescence when a karyotype was obtained as workup for delayed puberty. These are examples of the wide spectrum of clinical presentations and progression of disease that make the designations of Denys-Drash and Frasier syndromes indistinct. This contributes to the paradigm that calls for the genetic investigation including karyotype of any phenotypic female who presents with CNS, SRNS or proteinuria, or ESKD concerning for possible 46XY sex reversal associated with *WT1* glomerulopathy. The fact that the XY karyotype predominates in most series suggests that we are probably missing the diagnosis in a number of females, especially those with familial proteinuria (5, 8, 42, 43).

The risk of malignancy associated with *WT1 mutation* compels the discussion of whether prophylactic nephrectomy and/or gonadectomy should be considered once the diagnosis is confirmed. In the context of this study and others in the literature review, the characterization of the risks of malignancy according to phenotype–karyotype is extremely pertinent (14, 48, 49). Importantly, the current analysis of 333 cases shows that normal male XY phenotype–karyotype is at low risk for any malignancy. However, normal female XX phenotype–karyotype has very low risk for gonadoblastoma but remains at risk for Wilm's tumor, especially in early life. Historically, there has been an unsubstantiated concession to perform prophylactic nephrectomies in young children with *WT1 mutation*, especially in the setting of prior Wilm's tumor or in preparation for transplantation (48). Currently, there is no consensus for prophylactic nephrectomies. In the literature review, there were no specific recommendations in favor of prophylactic bilateral nephrectomy, and most recently, the recommendation has been for renal sparing surgery in the case of unilateral Wilm's tumor (14, 47–49). In marked contrast, there is a strong consensus for prophylactic gonadectomy in all phenotypic females with 46 XY sex reversal. Moreover, our results show the increased risk of malignancy in phenotypic males with DSD and emphasize the need for close surveillance and a low threshold for prophylactic gonadectomy (47–50).

Comprehensive data on the surgical and medical management of sexual health in children with *WT1 mutations* are lacking. There is a need to better understand hormonal replacement, erectile dysfunction, cosmesis for urogenital abnormalities, and reproductive capacity. Endocrine data are scarce in subjects with *WT1 mutations*. Long-term follow-up with the development of novel markers to evaluate and predict gonadal malignancies is imperative.

**Figure 4:**
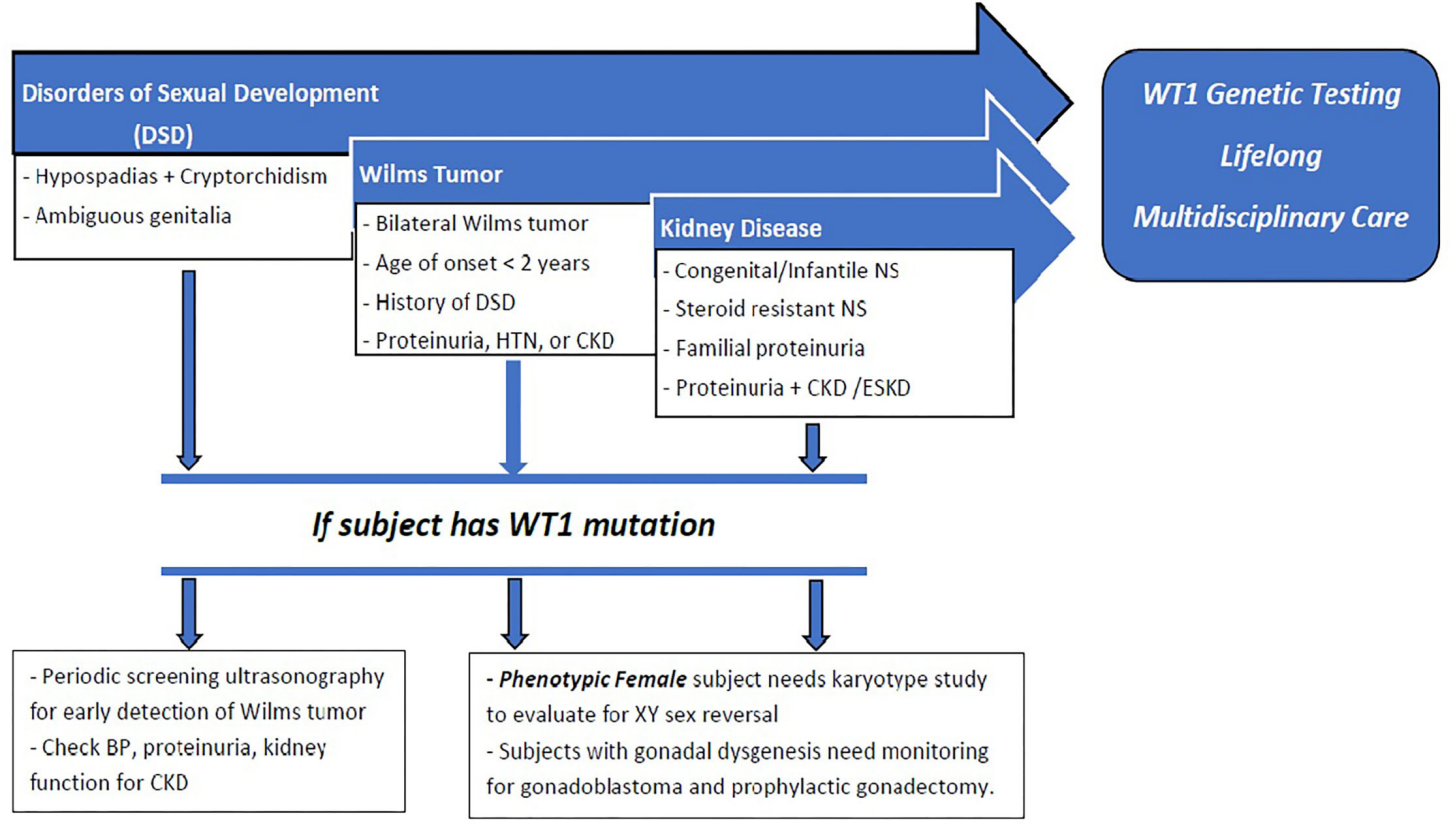
: Proposed paradigm for early recognition, comprehensive genetic testing followed by clinical surveillance of patients with *WT1 mutation*. Early presentation with DSD, CNS, SRNS, or Wilm's tumor should raise suspicion for *WT1 mutation*. Those with DSD should undergo periodic screening for Wilm's tumor, proteinuria, and development of CKD. All phenotypic females should have karyotype testing for possible 46XY sex reversal. All patients with *WT1 mutation* should receive long-term multidisciplinary medical, surgical, and psychosocial management. Phenotypic females with XY sex reversal should be closely surveilled for gonadoblastoma and recommended for prophylactic gonadectomy.

We propose a paradigm designed to initiate early comprehensive genetic testing followed by clinical surveillance (Figure 4). The goals are for (1) early phenotypic recognition leading to genomic diagnosis; (2) appropriate surveillance for progression to CKD, malignancy, and sexual health in patients with *WT1 mutations*; (3) genetic analysis to precede kidney biopsy in children with SRNS; (4) establish early medical management to modulate proteinuria and preserve long-term kidney function; (5) genetic counseling for the patient and family related to fertility and future pregnancies; and (6) expansion of the knowledge database by collaboration and reporting of new cases. Current recommendations include early genomic analyses by next generation sequencing (NGS) and karyotyping of any infants (< 1 year of age) with proteinuria including CNS, SRNS, and/or male DSD (51).

This study has several limitations including those inherent in extracting accurate detailed case characteristics from an in-depth literature review. There may have been publication bias from distinct case reports, particularly involving malignancy or karyotype–phenotype correlations. This is especially true in the case of XY-karyotype with severe gonadal dysmorphia and ambiguous phenotype. Nevertheless, the impact of *WT1 mutations* and the long-term consequences on patient's health merits comprehensive and contemporary review to promote early recognition and genetic screening.

## Conclusions

Early recognition of *WT1 gene mutation* is essential for appropriate medical and surgical management. This includes the use of renal preservation therapy to modulate proteinuria and hyperfiltration with angiotensin blockade, avoidance of immunosuppressive treatment before kidney transplantation, surveillance, and early detection of malignancy. Further, these patients would benefit from multi-disciplinary care from primary care providers, nephrologists, endocrinologists, urologists, and psychologists to establish appropriate long-term management of psychosocial and sexual health. In this case series, kidney transplantation has provided long-term patient and allograft survival with no increased incidence of malignancy post-transplant.

## Data Availability Statement

The original contributions presented in the study are included in the article/supplementary material, further inquiries can be directed to the corresponding author/s.

## Ethics Statement

The studies involving human participants were reviewed and approved by University of Miami Institution Review Board. Written informed consent from the participants' legal guardian/next of kin was not required to participate in this study in accordance with the national legislation and the institutional requirements.

## Author Contributions

PA-P, WS, and CA wrote the first draft of the manuscript. WS, CK, MD, and CA participated in the critical analysis of the data and the major revisions of the manuscript. All authors contributed to the conception, design of the study, contributed to manuscript revision, read, and approved the final version of the manuscript.

## Funding

This work was supported in part by the Lilian Barbarosh Pediatric Renal Research Fund.

## Conflict of Interest

The authors declare that the research was conducted in the absence of any commercial or financial relationships that could be construed as a potential conflict of interest.

## Publisher's Note

All claims expressed in this article are solely those of the authors and do not necessarily represent those of their affiliated organizations, or those of the publisher, the editors and the reviewers. Any product that may be evaluated in this article, or claim that may be made by its manufacturer, is not guaranteed or endorsed by the publisher.
